# Recent Developments in Polyurethane-Based Materials for Bone Tissue Engineering

**DOI:** 10.3390/polym13060946

**Published:** 2021-03-19

**Authors:** Piotr Szczepańczyk, Monika Szlachta, Natalia Złocista-Szewczyk, Jan Chłopek, Kinga Pielichowska

**Affiliations:** Department of Biomaterials and Composites, Faculty of Materials Science and Ceramics, AGH University of Science and Technology, Al. Mickiewicza 30, 30-059 Kraków, Poland; piotrszczepanczyk@gmail.com (P.S.); szm@agh.edu.pl (M.S.); zlocista@agh.edu.pl (N.Z.-S.); chlopek@agh.edu.pl (J.C.)

**Keywords:** polyurethane-based composites, bone tissue engineering, regenerative medicine

## Abstract

To meet the needs of clinical medicine, bone tissue engineering is developing dynamically. Scaffolds for bone healing might be used as solid, preformed scaffolding materials, or through the injection of a solidifiable precursor into the defective tissue. There are miscellaneous biomaterials used to stimulate bone repair including ceramics, metals, naturally derived polymers, synthetic polymers, and other biocompatible substances. Combining ceramics and metals or polymers holds promise for future cures as the materials complement each other. Further research must explain the limitations of the size of the defects of each scaffold, and additionally, check the possibility of regeneration after implantation and resistance to disease. Before tissue engineering, a lot of bone defects were treated with autogenous bone grafts. Biodegradable polymers are widely applied as porous scaffolds in bone tissue engineering. The most valuable features of biodegradable polyurethanes are good biocompatibility, bioactivity, bioconductivity, and injectability. They may also be used as temporary extracellular matrix (ECM) in bone tissue healing and regeneration. Herein, the current state concerning polyurethanes in bone tissue engineering are discussed and introduced, as well as future trends.

## 1. Introduction

The increase in life span forces regenerative medicine to look for new methods of treatment that can enhance regeneration of organs or tissues damaged by trauma or degenerative diseases. Tissue engineering, in particular, shows great promise [1]. According to the generally accepted definition, this domain applies the principles and methods of biomaterials engineering and medicine with a view to sorting out the structure–function relationship in both the pathological and healthy human tissue, and the design and fabrication of biological substitutes to improve, restore, or maintain, tissue function [2]. Scaffolds, cells and signalling biomolecules are the three basic components upon which tissue engineering is based [3]. Certain materials have been developed for bone regeneration, even though they currently do not conform to clinical standards. Clinically approved therapies for bone regeneration avail themselves of either autologous or heterologous demineralised bone despite several limitations, i.e., increased surgery time, insufficient availability, and significant morbidity linked to blood loss, wound setbacks, local sensory deficiency, surgical complications and, most importantly, chronic pain. In turn, allografts also possess osteo-inductive properties, but these can only be recognised if the graft is utilised in a demineralised form. Drawbacks of allografts include fracture, transplant rejection, the transmission of diseases, bacterial infection, and immunogenicity [4]. As a consequence of that, allografts must go through several treatments before use. Namely, they are devitalised by freezing and sterilised with ethylene oxide or γ irradiation, but all these sterilisation steps might notably cut down on the graft’s osteoinductivity. The steps include removal of soft tissue by means of ultrasonic baths and physical debridement. Lyophilisation and tissue freezing are used in the preservation of allografts, but this also substantially changes the material’s properties. Similar stages of inclusion by the host are observed as in the autograft, however, with the difference that the whole process is slower for allografts. This is caused by the lack of living cells which may be osteogenic and osteoinductive. Furthermore, the genetic differences between donor and organ recipients along with which the repair process is either slower or appears incomplete. This indicates that despite the preparation procedure, some immunogenicity remains unaltered. Xenotransplantation has shown poor clinical outcomes with graft rejection [5]. Therefore, engineered constructs could present an alternative source besides traditional transplants. Tissue-engineered implants consist of a two- or three-dimensional scaffold which acts as a carrier for biologically active cells and proteins that should stimulate the colonisation of the scaffold by host cells. To be applied in bone tissue engineering, scaffolds need to possess, above all, an interconnected open porosity where the pores are of an adequate size enabling chemotaxis, cell adhesion, proliferation, and differentiation [4,6]. Ideally, bone graft substitutes ought to be biocompatible, osteoconductive, biodegradable, osteoinductive, undergo remodelling, show minimal fibrotic reaction, support bone regeneration and new bone formation, and mimic a natural extracellular matrix as much as possible [3,7]. Considering the mechanical properties, synthetic bone graft substitutes should have similar mechanical properties to that of the bone being replaced. Especially, the modulus of elasticity should be at a similar level to that of bone to prevent stress shielding, over and above possessing adequate toughness. A similar modulus prevents fatigue fracture under cyclic loading. At present, synthetic, and natural polymers are promising materials for bone regeneration due to easy fabrication into three-dimensional structures, large available surface area for cell adhesion, proliferation, and migration, and controlled open porosity facilitating diffusion of nutrients and metabolism products. The most used naturally derived polymers in bone tissue engineering belong to alginates, collagens, hyaluronic acid, and gelatine [8,9,10]. As to synthetic biodegradable polymers, poly(α-hydroxyesters), polydioxanone, polyorthoesters, polyanhydrides, and some polyurethanes are most commonly exploited for bone tissue engineering [11,12,13,14,15,16,17,18,19,20]. However, it is quite difficult to attain an equilibrium between in vivo scaffold degradation and tissue regeneration due to the occurrence of many different clinical factors based on the geometry of the bone defect, and the functional loading influencing bone apposition and remodelling. Biointegration is another alternative factor to biodegradation. Polymeric scaffolds with a slow degradation rate are the benchmark. In this respect, polyurethanes are characterised by a broad range of mechanical, physicochemical, structural, and morphological properties, much broader than biodegradable polymers commonly used in the biomedical field [21,22,23,24]. Moreover, crosslinked polyurethane foams with controlled degradation rates, a proper and desired range of pore size, open porosity [21,25], different hydrophilicity [26], and a surface modified by protein coating [27] composites have been developed and characterised. Polymers are suitable carriers for biologically active factors among which polyurethanes constitute an important group [28,29,30,31,32,33,34]. The soft-to-hard segment ratio in polyurethanes’ structure plays a crucial role in determining the physical and mechanical properties which can be adjusted in respect of the desired application, varying the concentrations of each of the reactants and the synthesis procedure [35,36]. Consequently, they can be used in many areas of tissue engineering. Thanks to their good haemocompatibility, low friction rate and good mechanical properties, polyurethanes have been successfully applied as coatings on cardiac pacemaker electrodes, blood transfusion filters, and devices supporting physiological blood flow. There are also hydrogel burn dressings made of polyurethanes in common use [37]. Tissue engineering investigations have recently concentrated on the design and fabrication of bioactive materials that can interact with the surrounding tissues [38,39]. There are various strategies to improve material bioactivity. The most popular one is the incorporation of bioceramic fillers into polymer matrices [40]. As a matter of fact, biocompatibility is supposed to be the most important parameter of a bone tissue engineering scaffold. Once implanted, a scaffold is not to incite inflammation nor a toxic response. Therefore, not only the ultimate product, but also the unreacted substrates and released biodegradation products need to be biocompatible. It is also necessary to point out the significance of the osteoconductivity of biomaterials which allows bone cells to adhere, proliferate, migrate, and deposit bone. In order to induce both angiogenesis and bone growth inside a biomaterial, a microstructure with interconnected pores is to be expected, not to mention that it also provides an increased surface area available for cell attachment, growth, and function throughout a defect. Nowadays, injectable biomaterials are the most desirable for clinical applications because they can be moulded into irregular-shaped defects or directly injected in vivo and cross-linked in situ, thereby enhancing the implant–tissue contact and minimising the invasiveness of surgery. Both ceramic pastes and polymers including polyurethanes may be used in injectable formulations. Simultaneously, scaffolds are due to undergo sterilisation such that their bioactivity and chemical composition are not modified during processing [5,41]. As yet, polyurethane scaffolds are uniquely capable of being processed as an ideal bone tissue substitute. Their properties can be tuned for varied strength and elasticity.

## 2. Polyurethanes

Polyurethanes (PU) are polymeric materials that thanks to the possibility of modifying their structure or method of preparation, have a very wide range of both chemical and physical properties. As a result, they can be adapted to the various requirements of current technologies such as fibres, adhesives, coatings, thermoplastics, and thermoplastic elastomers [42].

**Figure 1:**
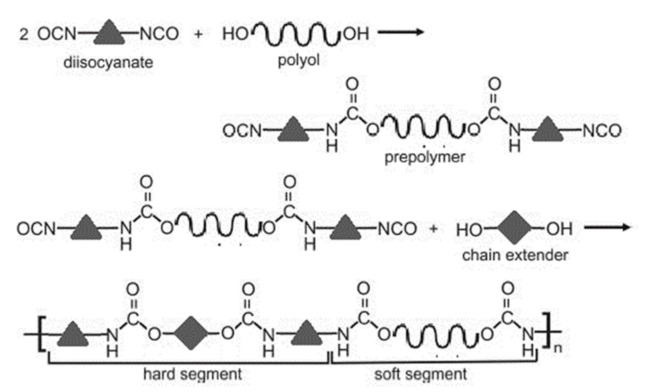
Mechanism of PU synthesis.

The polyurethane synthesis is the polyaddition reaction with a polyester or polyether polyol and a chain extender (Figure 1). A segment polymer is obtained in which the hard and soft segments can be distinguished.

The hard segments formed from chain extenders/crosslinkers and the isocyanate are stiff, immobile, and often hydrogen bonded, while the soft segments formed from oligomer polyols are usually present in a coiled formation, or can crystallise and are a mobile and soft phase in polyurethanes [43]. Substrates usually used for polyurethane synthesis are presented in Table 1.

The nature of the chemistry allows the production of polyurethanes as different types of materials, like thermoplastic elastomers or flexible and rigid foams. Biomedical polyurethane (PU) elastomers, due to their durability, toughness, biocompatibility, good biostability, and even bioactivity, have been applied in many biomedical devices. The properties, stability and morphology of PUs are dependent on the microphase segregation between the soft and hard segments. The presence of strongly polar groups in the rigid segments significantly improves the polarity, intermolecular forces, and hydrogen bonding within those segments. This results in an increased microphase separation, a higher glass transition temperature and melting point of the hard segments, and thereby, an enhanced mechanical strength of the biomaterial [44]. Conversely, biomaterials used in tissue-engineered scaffolds are often designed to biodegrade in the human body and assist in the formation of functional tissue. In recent years, biodegradable PUs have become more desirable for use as tissue scaffolds and drug delivery systems. The biggest advantage is the broad range of physicochemical properties that can be achieved through different substrates. Moreover, PUs can be prepared by reactive injection moulding (RIM), thus make them potentially suitable as injectable biomaterials for noninvasive therapies [45]. This review presents recent advances in the development of PUs for applications in bone tissue regeneration and replacement.

## 3. PU-Based Materials in Biomedical Applications

Biomedical devices made of biostable PUs were used in the 1960s for the first time. Alternatively, biodegradable PUs have recently been examined for biomedical applications, especially in regenerative medicine and tissue engineering. In contrast to the long-term biostable implants, biodegradable PUs are specially devised to undergo controlled, gradual degradation in vivo and to enhance ingrowth of the new tissue. Techniques for fabrication of biodegradable tissue-engineered scaffolds include:

- thermally induced phase separation,
- salt leaching,
- wet spinning,
- electrospinning,
- carbon dioxide foaming,
- 3D printing.

Tunable properties make biodegradable polyurethanes useful for regeneration of tissue. PU elastomers with improved hydrolytic stability have recently been developed [45,46,47]. It should also be noted that the secretion of biologically active molecules by activated macrophages can enhance the biodegradation of PU implants and affect their biostability. Scientists, at one point, realised that the PU degradation products included toxic, carcinogenic, and mutagenic aromatic diamines [48]. Synthetic biodegradable polymers like poly(α-esters), l-tyrosine derivatives, poly(propylene fumarate), and polyphosphazenes are known to support cell attachment and proliferation. They also biodegrade to noncytotoxic products; hence, they have been incorporated in drug delivery systems and scaffolds. An advantageous aspect of PU chemistry is the broad range of biological and physicochemical properties (Table 2).

This paper presents the latest advances in tissue engineering based on biodegradable PU. Scaffolds for the repair of the musculoskeletal, neurological, and cardiovascular systems have been discussed most widely [47,71,72] in the next three subsections.

### 3.1. Scaffolds Designed for Cardiovascular Applications

**Figure 2:**
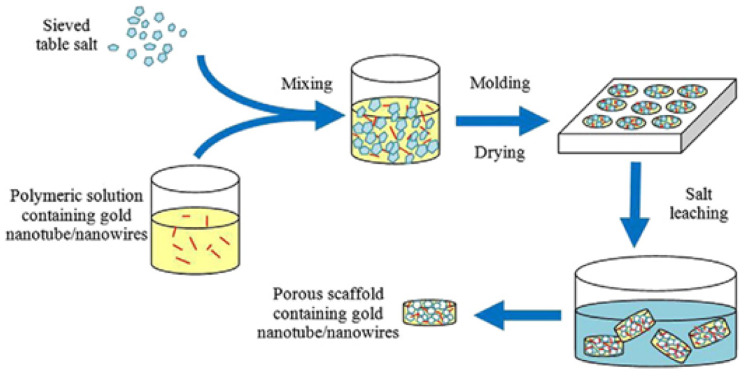
Schematic of the scaffold preparation. Reprinted from [49] with permission from Elsevier.

For cardiovascular tissue repair, materials with high elongation at break, high tensile strength, biocompatibility, and biodegradability are required. It has been noted that the required properties are exhibited by PU elastomers made from lysine ethyl ester, 1,4-diisocyanatobutane (BDI), or PCL-*b*-PEG-*b*-PCL or PCL macrodiols, and from putrescine chain extenders [50,51,73,74]. The materials were soft elastomers, showing elongation at break from 325% to 895%, and having a tensile strength of 8–29 MPa. In vitro studies have shown that these materials support endothelial cell adhesion, and, in addition, their degradation products show no sign of cytotoxicity. A porosity of 80–97% was obtained due to the use of thermally induced phase separation using dimethyl sulphoxide. Modifications of the polymer concentration in solution, or quenching temperatures, led to the formation of random or oriented pores. It has been noted that PCL-based materials exhibited slower hydrolytic degradation and smaller smooth muscle cell growth compared to scaffolds based on PCL-*b*-PEG-*b*-PCL block copolymers. Nanofibers based on type I collagen, and PU produced by electrospinning, showed good strength properties, and supported enzymatic and hydrolytic degradation, and cell adhesion. These materials are good flexible regenerative matrices [75]. By combining the mechanical properties of polyurethane elastomers with collagen, a biomimetic material modelled on the extracellular matrix was obtained. PU elastomers obtained by electrospinning, and constructions produced by electro-spraying of smooth muscle cells, have comparable mechanical properties to those of native arteries [73]. Segmental PU elastomers have been synthesised to improve the enzymatic degradation of the hard segment. The material was obtained by synthesis of a diester diamine chain extender containing l-phenylalanine, a cyclohexanedimethanol adduct, and a soft PCL segment of macrodiol and lysine diisocyanate was used as the second product. Due to its almost complete resorbability after only 12 weeks and supporting cell adhesion, PU is a promising material for use as a cardiovascular scaffold. PU was examined for a heart patch [74] or a cell supply system that would strengthen the heart after a heart attack [76,77]. Myocardial infarction may cause failure due to cardiomyocyte loss. Studies were carried out on Lewis rats using a biodegradable, flexible PU patch that was designed to promote ventricular remodelling and improve their function. After analysing the research, it was concluded that the patch promotes the formation of new tissue suggesting that it is a potential alternative method to repair the heart. By incorporating gold nanoducts/nanotubes (GNW/NT), biodegradable nanocomposites with a wired structure were synthesised (Figure 2). Thanks to this solution, such material can mimic the electromechanical properties of the heart. Studies have shown that modified PU nano-silver has shown better fibroblast adhesion and better physicochemical properties [78]. The advantage of gold in the form of nanowires and nanotubes is the formation of bridges between pores in the polymeric structure, which improves cell communication [49].

Chiono et al. [79] prepared bilayered scaffolds using melt-extrusion additive manufacturing (AM) for PU with PCL-based soft segments, 1,4-BDI and l-lysine ethyl ester dihydrochloride chain extender. The obtained scaffolds showed highly reproducible computer-aided design geometry and exhibited an elastomeric-like behaviour that is desired in myocardial applications

### 3.2. Scaffolds Designed for Musculoskeletal Applications

Fibres based on MDI, 1,3-diaminopropane and PCL diol exhibit high tensile strength and maintain enough high tensile strength under degradation and could have found application for the replacement of anterior cruciate ligaments. The commercial application of these materials has been effectively exploited by Artimplant and called Artelon^®^. After implantation in rabbits for anterior cruciate ligament reconstruction, the mentioned fibres did not show mutagenic effect and fatigue subsequently to repeated cyclic loading. The ingrowth of connective tissue was established after 6 months from implantation. As a result of 24 months of implantation, the degradation of the polymer fibres was detected. Inflammatory and unwanted joint reactions were not proved, although macrophages and multinuclear giant cells were present [80].

Biodegradable scaffolds prepared from segmented PU elastomers have been studied for their potential application in healing of the knee-joint meniscus in dogs. They were synthesised from butane 1,4-diisocyanate (BDI) and a 50/50 poly(ε-caprolactone-co-l-lactide) diol to attenuate the potential risk of releasing toxic aromatic diamines. They displayed both interconnected macropores with 150–300 mm size, and micropores smaller than 50 mm, and had a compression modulus of 200 kPa which is suitable for the regeneration of fibrocartilage [81].

Segmented PU elastomers which indicate biodegradable features have demonstrated the ability of the regeneration of bone tissue as a scaffold. The synthesis of diurea diol chain extenders has been successfully conducted by the reaction of 2 mol of tyrosine or tyramine with 1 mol of 1,4-diisocyanatobutane (BDI). This process enabled the mimicry of MDI-based hard segments in the obtained polyurethane. The application of these chain extenders has made a significant contribution and allowed the synthesis from aliphatic diisocyanates and a polyethylene glycol macrodiol as the soft segment. The presence of phenyl groups in the tyramine and tyrosine moieties in the backbone is suitable to give rigidity to the hard segment. The development of tyramine- and tyrosine-derived polymers results in the in vitro attachment and proliferation of viable MG-63 human osteoblast-like cells. To analyse the impact of hard segment loading on the biological and physical properties, biodegradable segmented PU elastomers were obtained from BDI and the tyramine-based chain extender and PCL macrodiols, with an average molar mass from 1100 to 2700 g/mol. It was observed that increasing the PCL molar mass caused an increase in the melting temperature of the soft segments from 21 to 61 °C, and the storage modulus from 52 to 278 MPa. Results suggest that the crystallisation of soft segments significantly changes the mechanical properties of PU elastomers. Bone marrow stromal cells cultured on PU films in osteogenic medium were similar for each of the PU films and the poly(_D,L_-lactide-co-glycolide) positive control. Scaffolds fabricated from PU elastomers obtained using HDI, different macrodiols as soft segments, and different chain extenders have been revealed to support bone healing in iliac crest defects in sheep. The content of the hydrophilic soft segment comprised a PEG or a poly(ethylene-*b*-propylene-*b*-ethylene oxide), which was varied from 30% to 70%, wherein the hydrophobic soft segments were a PCL-diol. As chain extenders, BDO, 2-amino-1-butanol, 2-mercaptoethylether, and isosorbide diol were investigated. Porous scaffolds with porosities of up to 90% were prepared using a salt leaching/phase inversion method. By incorporating β-TCP or HAp, the osteoconductivity and other properties of the materials were improved. It was found that the ability for mineralisation and calcification increased with increasing hydrophilicity. Moreover, the rate of mass loss during investigation of in vitro chemical stability showed that poly(ester ether urethane)s degraded faster than poly(ether urethane)s. After 6 months, new bone with a Ca/P ratio similar to that of healthy bone had grown into the pores of the PU scaffolds, but the newly formed bone exhibited a higher mineral content in the more hydrophilic PU.

**Figure 3:**
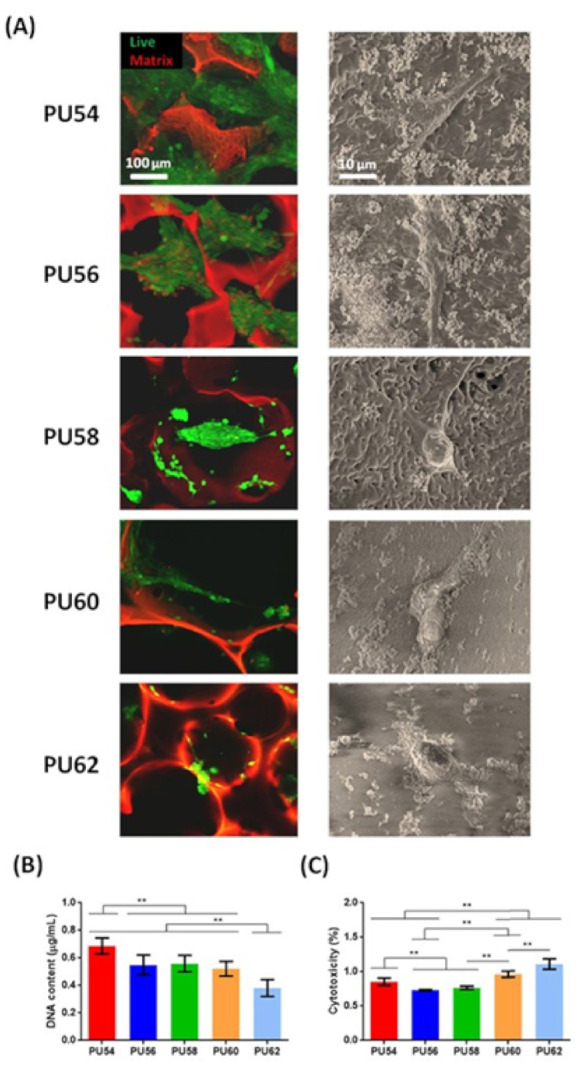
(**A**) Confocal microscopy images (**left**) and correspondent SEM images (**right**), of C2C12 skeletal muscle cultured on PU-based scaffolds. In fluorescence images, live cells are shown in green, while the scaffold matrix is shown in red. (**B**) DNA quantification for C2C12 skeletal muscle cells on each sample type, 24 h after seeding. (**C**) Cytotoxicity expressed as the ratio between the lactate dehydrogenase (LDH) released from cells cultured on the samples and the one released from a positive control featured by relevant toxicity, 24 h after seeding. Reprinted from [64] with permission from Elsevier.

Vennozzi et al. [64] investigated soft PU-based 3D porous scaffolds with different mechanical properties for skeletal muscle cells. In in vitro tests they observed better adhesion of skeletal muscle cells over the softer and more porous structures (Figure 3).

### 3.3. Scaffolds Designed for Nerve and Other Soft Tissues Regeneration

Autologous nerve grafting is considered as the golden standard in nerve defects, but the limited source of autologous nerve grafts impedes nerve damage therapies. Hausner et al. [82] investigated biodegradable PU tubular fibrin coated scaffolds. The aim was to investigate a resected segment of rat sciatic nerve. According to the investigation results, biodegradable PU tubular scaffolds support peripheral nerve regeneration. Animals treated with tubular scaffolds had no significant functional differences compared with the control group. No sign of decomposition could be seen 3 months after implantation.

Biodegradable block PUs have been applied in nerve tissue engineering. Porous scaffolds were obtained from PCL-diol, PEG and HDI, using the dipping-leaching method. The authors found that PU nerve scaffolds are characterised by a better regeneration ability than PCL or silicone, and comparable to that of autograft. Moreover, quite good electrophysiological recovery was observed: 76% and 87% of rats for PUCL-*ran*-EG and autograft, respectively. Biodegradation of block polyurethane nerve scaffolds occurred until 20 weeks in vivo and until 16 weeks in vitro, and PUCL-*ran*-EG nerve scaffolds could help with peripheral nerve regeneration [83].

The double-nozzle, low-temperature, deposition manufacturing system is based on a digital prototyping approach and combines both thermally induced phase separation and freeze-drying. Cui [84] used the above mentioned technique to prepare double-layer polyurethane/polyurethane-collagen nerve channels for peripheral nerve regeneration. Optimisation of the preparation parameters and biomaterial concentrations were directed to ideal nerve repair tubes composed of internal oriented filaments of collagen, and an outer microporous layer of PU.

Biodegradable nerve guidance channels have been synthesised from alternating block copolymers comprising semicrystalline poly[(R)-3-hydroxybutyrate] (PHB)/HV, and amorphous poly[glycolide-co-(ε-caprolactone)]-diol (PGC-diol), linked by an LDI and 2,2,4-trimethyl hexamethylene diisocyanate (TMHDI). Investigations showed the ability of these materials to regenerate nerves. The materials also turned out to be compatible with cells and tissue both in vitro and in vivo [85].

Poly(ester urethane)urea (PEUU) and poly(ether ester urethane)urea (PEEUU) have been synthesised from PCL, PCL-*b*-PEG-*b*-PCL, BDI, and putrescine. The porosity of the obtained scaffolds ranged from several µm to more than 150 µm. PEEUU exhibited lower mechanical parameters than PEUU. Biodegradation in phosphate buffered saline (PBS) varied with the PU hydrophilicity and porosity. Both types of PU support cell adhesion, proliferation, and growth, but the PEEUU scaffold allowed cells to grow more extensively. Soft tissue includes ligaments, tendons, skin, fascia, synovial membranes, fibrous tissues, and fat (which are connective tissue), and blood vessels, nerves and muscles (which are not connective tissue) [65,86].

Another example of prospective materials for soft tissue engineering are biodegradable PU blends made from PEG, PCL, amino acid-based chain extenders, and diisocyanates. The PEG material turns out to increase the blend’s susceptibility to degradation, while the PCL-based parent PU provides higher moduli. Different soft segment compositions allow the production of a family of semicrystalline, elastomeric materials with a wide range of morphological, mechanical, and degradative properties. They can also be formed into 3D porous scaffolds utilising solvent casting/particulate leaching methods [87].

Block polyether-based PUs are considered as partially biodegradable polymers because of their polyether moieties. Wu et al. [88] used poly(glycerol sebacate) (PGS), aniline pentamer (AP), and hexamethylene diisocyanate (HDI), to prepare conducted polyurethane films. Poly(glycerolsebacate-*co*-aniline pentamer) (PGSAP), obtained via polycondensation, was crosslinked by HDI. The mechanical properties of PGSAP polyurethanes were changed by different AP contents. An increase of AP amount caused an enhancement of the Young’s modulus from 1.5 to 75.5 MPa, respectively. These results show that PGSAP polyurethane could be successfully used for numerous advanced applications in soft tissue engineering. The polyurethane film prepared in this way has been employed in peripheral nerve regeneration, leading to neurite growth and elongation.

More recently, Grzesiak et al. [24] described a polyurethane (PU) and polylactide (PLA) blend designed for the regeneration of the olfactory bulb-derived glial cells. The biomaterial involved PLA, and PU made of PCL, HDI, and isosorbitol as a chain extender. This led to improvements in the size and the number of pores in the material that could regulate the degree of cell adhesion. The obtained results of Young’s modulus showed that the incorporation of PLA in a PU/PLA blend caused an increase in the tensile strength compared to the pure PU.

## 4. Bone Tissue

Bone tissue is characterised by a good healing ability and the majority of bone defects heal spontaneously, stimulated by proper biological and micro-environmental conditions. Throughout life, bone tissue undergoes an active continuous process of resorption, remodelling, and formation with the aid of the osteoblasts. High-magnitude and high-rate strains provide the stimulus for the adaptation of bone, so the load-bearing role of the bone is unceasingly optimised by the remodelling activity. Adaptation appears at the structure of an organ entailing changes in the bone architecture. Increased load application stimulates bone formation [89,90]. In turn, bone tissue is removed from the skeleton in response to lower loading [91].

Cellular signalling in bone consists of lining cells, osteoblasts, osteoclasts, and osteocytes. Apart from this, haematopoietic and mesenchymal progenitors play a crucial role. The bone remodelling stages are strictly controlled by communication between the bone cells. Osteocytes enhance lining cells on the bone surface that subsequently produce cytokines to recruit osteoclast progenitors to the bone surface. Stimulation of mature osteoclasts causes creation of a resorption pit. Then, factors contributing to the adhesion, proliferation, and differentiation of osteogenic progenitors into mature osteoblasts, are unleashed. Osteoblasts yield osteoids to which some osteoblasts are anchored, and as a consequence of mineralisation of the matrix, they become osteocytes. Remaining osteoblasts evolve into lining cells or die out. Growth factors, amongst others important in bone signalling, are bone morphogenetic proteins, the wingless-type MMTV integration site family of proteins, parathyroid hormones, hedgehogs, and insulin-like growth factors. In bone gender steroids, oestrogen and androgen also affect bone metabolism through attachment to intracellular receptors. Having bound, they inter-react with binding elements in promoter regions. Lack of these gender steroids brings on osteoporosis by the considerable augmentation of secreted signalling molecules that regulate bone resorption [92]. Vitamin D is another factor with pronounced effects on bone [93].

The minerals and tissues present in bone ensure that it has a perfect loading and tensile strength. The stiffness of bone is dictated by the occurrence of the inorganic phase of bone containing minerals [93]. Cells and tissue form the organic constituents are necessary for the upkeep of the elasticity and tensile strength. Osteocytes are the basic type of cells found in mature bone tissue. They are located in osseous lacunas lying within the bone plates with a slightly looser fibre arrangement, surrounded by a completely mineralised part of the bone. The mineral matrix of bones was created as a result of deposition by the osteoblasts of a noncalcified matrix which undergoes calcification by deposition of calcium salts. Osteocyte processes occur in the bone tubules surrounded by a nonmineralised intercellular matrix. The osteoblasts occur where new bone is created. Part of the osteoblasts transform into osteocytes, and the rest go into resting, stadium-like, flat-bone-lining cells. The osteoprogenitor cells located in the bone marrow and membranes lining both the inner and outside surface of the bone, produce active osteoblasts. Bone contributes to the regulation of calcium and phosphate blood concentrations as well. The osteoclasts, the osteocytes, and the osteoblasts are crucial in this process. Finally, bone contains the bone marrow where the white and red blood cells stem from [94].

**Figure 4:**
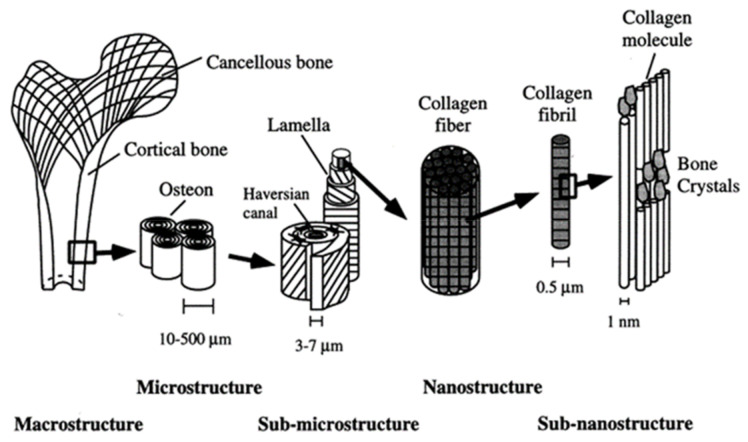
Hierarchical structural organisation of bone: (left to right) cortical and cancellous bone; osteons with Haversian systems; lamellae; collagen fibre assemblies of collagen fibrils; bone mineral crystals, collagen molecules and noncollagenous proteins. Reprinted from [96] with permission from Elsevier.

In general, the skeleton consists of long bones, flat bones, and cuboid bones. Structurally, two basic types of bone are identified: cancellous and cortical bone. With a higher ability for change and activity in growth, haematopoiesis and calcium homeostasis have been found in cancellous bone. Its supportive function predominates in locations with a compressive type of loading, like in vertebra and adjoining to articulated joints. Compact bone is rigid and does not undergo alterations. It is located, for the most part, in the shafts of long bones and also in the flat bones. In the microscale, the woven and lamellar bones can be identified. Woven bone is the one where the ossein fibres are typically arranged in an unorganised pattern. Woven bone is produced very quickly by the osteoblasts and it is much weaker than lamellar bone. Woven bone helps with the repair of a fracture until it can be replaced by stronger material, and it is present in newborns as well. Woven bone also occurs in locations where fast bone creation takes place. In time, woven bone undergoes remodelling to lamellar bone. Lamellar bone is mechanically strong and has a regular parallel alignment of collagen into sheets. Haversian channels are surrounded by lamellar bone (Figure 4). Most of the bones in an adult’s body are made of lamellar bone [95].

Calcification can take place in extracellular bone matrix but also in artery walls. Ossification is exclusively the calcification/mineralisation of osteoid. It is predominantly constituted of collagen type I fibres. It is widely accepted that osteoid calcification is launched by the creation inside the matrix of small buds playing a role as nucleation sites for collagen fibrils and the initial stage of collagen mineralisation. Alkaline phosphatase hydrolyses calcium and phosphate containing organic substrates to increase mineral deposition. No naturally occurring substances with inhibitory effects contribute to inappropriate ectopic calcification at extra-skeletal sites. Specific processes such as necrosis and inflammation are related to decreasing the activity of these inhibitors which results in local calcification [97].

Formation, maintenance, and repair of bone goes ahead through intramembranous and endochondral mechanisms. Flat bones are built up through intramembranous bone formation followed by condensation of loose mesenchymal tissue containing osteogenic cells. The ossification begins in conjunction with appropriate vasculature, and then proceeds without any intermediate. Spongy bone is formed by trabeculae which, in turn, are built of spicules—small masses of bone. On the trabecular surfaces a so called appositional bone formation takes place which consists of osteoid formation, layer upon layer. This process keeps on until sufficient bone density is achieved. Then bone resorption and bone formation are active simultaneously in order to gain the most optimal shape and density. Thus, either compact or cancellous bone might be formed. Though intramembranous bone formation is highly productive, it is improper for the quick longitudinal bone growth of arms and legs in childhood. Long bones are formed through an endochondral bone mechanism which is preceded by the formation of cartilage. Mesenchymal cells condense and differentiate into chondroblasts that leads to formation of a cartilage matrix which covers the bone tissue with a dense fibrous layer (perichondrium). At the beginning, lengthening consists in the arrangement of repeated chondrocyte divisions and matrix production. Widening of the limb bud, in turn, is carried out by appositional growth from the perichondrium. Then, ossification centres appear in bone, first centrally and next at the extremities. Ossification is also connected with increasing of the intracellular calcium concentration. The thin cartilage matrix is a substrate for the calcification process that coincides with the chondrocytes apoptosis [41]. Thus, calcified scaffolding forms for later bone apposition. Simultaneously, the perichondrium differentiates into periosteum which is a source of preosteoblasts. Then osteogenic cells move into the calcified cartilage thus coercing the osteoid into deposition. Almost the entire bone is ossified excluding the central canal and two transverse plates located beneath the epiphysis, which are responsible for bone elongation until adolescence.

Bone grafting can be a successful therapy in cases where the bone repair mechanism fails as a result of magnitude, infection, or tumorous growth. Complicated fractures or severely atrophied regions need a bone graft. Several mechanisms of new bone formation and regeneration have been identified. However, ways in which a bone graft can help repair bone are still unknown. The provision of scaffolding that allows deposition of bone followed by integration with surrounding bone are crucial for the healing process. Osteoconductivity is the feature of a material which supports the attachment of new osteoprogenitor cells and osteoblasts. Grafting provides a 3D structure with open porosity through which new cells can migrate, and new blood vessels and nerve connections can form. Osteo-induction means that factors contained in the extracellular matrix can induce encircling nonbone cells to differentiate towards osteoblasts. Osteogenesis consists in the formation of bone thanks to the presence of osteogenic cells. Vascularised grafts enable osteogenesis which is a key in very large defects. Bone grafts should also be resorbable and not interfere with physiologic bone adjustment. The autologous cancellous bone graft possesses all of the above qualities. That is why it poses the gold standard. However, it carries limited mechanical strength that is sometimes desired. Accordingly, more dense cortical bone might be transplanted at the cost of the other qualities. Grafting may be indicated for nonhealing fractures, especially when the stability may be sufficient but the biology is lacking [98].

## 5. PU-Based Materials in Bone Tissue Engineering

### 5.1. Polyurethane Elastomers and Foams

Segmented PU elastomers have been studied as potential scaffolds for bone regeneration. The chain extenders were synthesised by coupling tyramine or tyrosine with BDI to reflect the structure of MDI-based hard segments. As a result, PU was synthesised from aliphatic diisocyanates (BDI and LDI) and the macrodiol PEG segment. Phenyl groups in tyramine and tyrosine are responsible for the rigidity in the skeleton. All polymers derived from these two compounds support both proliferation and attachment of osteoblast-like cells [99]. HDI-based PU, soft macrodiol segments, and various chain extenders have shown support in the healing of the iliac crest bone in sheep. In the soft segment, the hydrophilic content varied in the range of 30–70%, the hydrophilic component contained poly(ethylene-b-propylene-b-ethylene oxide) or macrodiol PEG, and the hydrophobic component was polycaprolactonediol. 2-mercaptoethyl ether, 1,4-butanediol, isosorbide diol, and 2-amino-1-butanol, were used as chain extenders [85,100]. Using salt leaching or phase inversion, scaffolds of up to 90% porosity were prepared, mainly from segmented polyurethane elastomers. The introduction of bioceramics in the form of HAp and TCP improved the material’s osteoconductivity. After conducting in vitro tests, both calcification and hydrophilicity increased, as well as an increase in all mineralised materials. After in vitro ageing, the weight loss rate was tested. The obtained speed results were higher for poly(urethane esther) than for poly(urethane ether). After implantation of the PU porous scaffold into the Iliac comb, healthy sheep had new growth of the spongy bone. Studies after 6 months in a group of examined sheep showed that the new bone produced showed a ratio of calcium to phosphorus comparable to the ratio of these elements in healthy bone. In addition, it was noted that when more hydrophobic implants were used, the bone showed a lower mineral content. For all materials only soft tissue ingrown on the surface of the defects was produced, and not, as expected, by the new cortex. For sheep aged 18–25 months having an oestrogen deficiency, it was observed that scaffolds with a calcium complex compound gave the best effects of bone regeneration. Hydrophilic scaffolds with vitamin D3 showed the worst bone regeneration. The researchers associated this with a too fast material degradation [85].

The first class of these biodegradable PUs used aromatic diisocyanates in their synthesis. However, it turned out that, through hydrolysis, these aromatic components can be transformed into potentially carcinogenic diamines.

**Figure 5:**
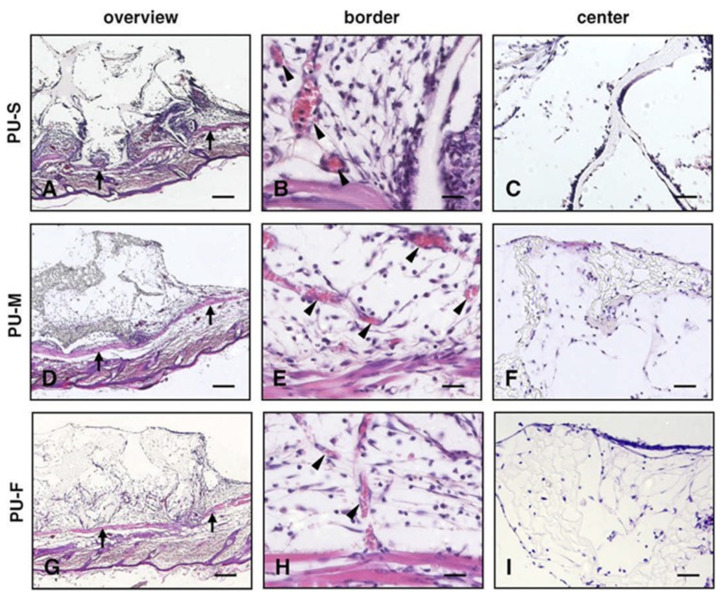
H&E-stained cross-sections of the PU-S (**A**–**C**), the PU-M (**D**–**F**) and the PU-F (**G**–**I**) scaffold at day 14 after implantation onto the striated muscle tissue (**A**,**D**,**G**, arrows) of the dorsal skinfold chamber. Higher magnification of the basis of the scaffolds (**B**,**E**,**H**) shows a newly formed granulation tissue in the border zone growing into the pores of the scaffolds. Within this granulation tissue, newly formed blood vessels (arrowheads) can be observed. In contrast, the centre of the scaffolds (**C**,**F**,**I**) is still avascular with only a few single cells migrating along the polyurethane strands. Scale bars: A, D and G = 220 lm; B, C and E = 25 lm; F, H and I = 55 lm. Reprinted from [102] with permission from Elsevier.

However, it is possible to replace the aromatic isocyanates in the urethane hard segments with aliphatic diisocyanates. Spaans et al. [66] investigated PUs based on 1,4-butanediisocyanate which hydrolyses to butanediamine, which is a normal component of all mammalian cells. It also plays an important role in the regulation of cell growth. The structure of PU consists of [A-B]n block pairs covalently linked in an alternating sequence. The soft segments A, derived from PDLA or PCL diols, are amorphous or slightly crystalline and able to hydrolyse with a glass transition temperature below body temperature. The hard segments B, derived from aliphatic BDO or BDI, have a melting point above body temperature. The flexible and elastic behaviour is connected to the soft segments and hydrogen bonds crosslinking between the hard segments and soft segments in a three-dimensional rigid network. In contrast to the soft segments, the hard segments have a uniform length which enhances the formation of hydrogen bonds that leads to an improvement of the mechanical properties, and the material behaves like a cross-linked elastomer. The physically cross-linked network is reversible as opposed to a chemically cross-linked one. Generally, polyurethanes are easily processable by extrusion, solvent casting, or freeze drying. By either cooling down the material, or removing the solvent, both the cross-linked network and its properties can be restored. Biodegradability in vivo is one of the key features, however, more appropriate to the investigation of the degradation process and the degradation paths and products, are in vitro studies, and also the accelerated degradation protocols. PUs based on BDI underwent degradation both at a physiological temperature and 60 °C. In vitro results showed that PU scaffolds remained intact for a sufficient time period to be useful for native tissue regeneration. The biodegradation rate of PU depends on the temperature, and it is lower at lower temperatures. Investigation of biodegradation at higher temperatures can be helpful e.g., to compare the initial degradation step. However, it should be noted that the in vivo biodegradation behaviour cannot be forecast as the properties and composition of the remaining products of biodegradation are different from degradation at 37 °C. Total biodegradation and bio-resorption needs to be investigated in additional long-term in vivo studies, but some parameters like biostability, the initial mass loss rate, as well as the thermal properties, can be studied with in vitro studies [101] (Figure 5).

Biodegradable poly(urethane-urea)s (PUU) elastomers are ideal candidates for different tissue scaffolds with selected properties similar to soft tissues such as muscle, cartilage, or bone. PUUs with semicrystalline PCL diol soft segments show good resilience and elasticity at small strains. Under larger strains their resilience is poor which has been explained by stress-induced crystallisation of the PCL segments. Amorphous poly(trimethylene carbonate) PTMC or poly(δ-valerolactone-co-ε-caprolactone) PVLCL, of different molar mass, make PUU softer and more resilient. Mechanical properties of the PUUs were determined by tensile testing. All of the obtained PUUs exhibited high elongations at break in the range 800–1400%, and also a high tensile strength from 30 to 60 MPa. PUUs with amorphous segments showed enhanced resilience and elasticity in comparison to the PUU with semicrystalline PCL-based soft segments, especially for the PUUs with a higher molar mass of soft segments. The soft segment molecular weight is inversely proportional to the tensile strength. Accelerated degradation experiments in phosphorus buffer saline with lipase concentration of 100 U/mL exhibited a higher mass loss for PCL-based PUUs than in PVLCL-based PUUs. PVLCL also degraded to a larger extent than PCL containing PUUs. The different kinds of synthesised PUUs showed variable elastomeric behaviour that could be unravelled on the basis of molecular design and crystalline behaviour [103].

A family of biodegradable PEUURs were synthesised using BDI and PCL diols of a mass-average molar mass ranging from 1100 to 2700 Da. As a chain extender, the prepolymer tyramine (TyA) was used. The hard segment content was 20–40%. Physical characterisation of the resultant polymers involved DMA, DSC, WAXS, and contact angle goniometry. DMA investigations showed storage moduli in the range of 49–278 MPa at body temperature, while DSC and WAXS results showed the correlation between the soft phase crystallinity and the storage modulus. Bone marrow stromal cells cultured on samples showed cell densities and other parameters similar to PLGA [23].

Poly(PHB/PEG urethane) copolymers with short PHB segments were obtained and investigated by Liu et al. [104]. The authors obtained poly(PHB/PEG urethane)s with low PHB crystallinity using the block copolymerisation method. The copolymers low PEG content ensured stability of the electro-spun fibrous scaffolds incubated in body fluid. The optimum PEG content also resulted in enhanced plasticity in the hydrated state [104].

Zhang et al. [105] prepared LDI-based PUs as potential materials for biomedical applications. The prepolymer was synthesised using highly purified LDI made from the lysine diethylester and glycerol. Afterwards, the LDI glycerol prepolymer was chemically foamed using water as a foaming agent, with the liberation of CO_2_. It was found that PUs obtained from LDI and glycerol can also be foamed in aqueous solutions, the foam undergoing degradation in aqueous solutions with nontoxic products being formed. In vitro investigation showed that this kind of scaffold supported growth and phenotype of bone marrow stromal cells (BMSC) over a period of 30 days. Eglin et al. [106] invented new biodegradable biocompatible PUs based on aliphatic diisocyanate, PCL diol, and 1,4:3,6-dianhydro-dsorbitol(isosorbide diol) as a chain extender. Such scaffolds are characterised by an improved affinity toward cells and tissues. The polymer was processed into three-dimensional porous foamed scaffolds with controlled pore size distributions using a combined salt leaching-phase inverse method. The scaffolds are expected to be applied as cancellous bone graft substitutes.

Laschke et al. [102] analysed the in vivo host tissue reaction to porous PU scaffolds based on HDI, PCL diol and different chain extenders: 1,4,3,6-dianhydro-D-sorbitol (PU-S), bis(2-mercaptoethyl) ether (PU-M), and 1,4,3,6-dianhydro-D-sorbitol with 3,7,11-trimethyl-2,6,10-dodecatrien-1 diaminobutane amide (PU-F). The PU-S scaffold with a compressive stiffness in the range of 22–145 kPa was applied for cartilage regeneration. The mechanical properties of PU-M are similar to those of PU-S. The presence of thiourea groups, more sensitive to hydrolysis than the urethane groups, makes the scaffold more sensitive to biodegradation. The authors also proved that isoprenoid molecules could positively influence cell viability and morphology, which was utilised in synthesis of the PU-F. The compressive stiffness of the scaffold was in the range of 10–57 kPa. The obtained results demonstrated that the porous PU scaffolds PU-S, PU-M and PU-F show good biocompatibility, and can be applied in tissue engineering without a strong inflammatory response [102].

**Figure 6:**
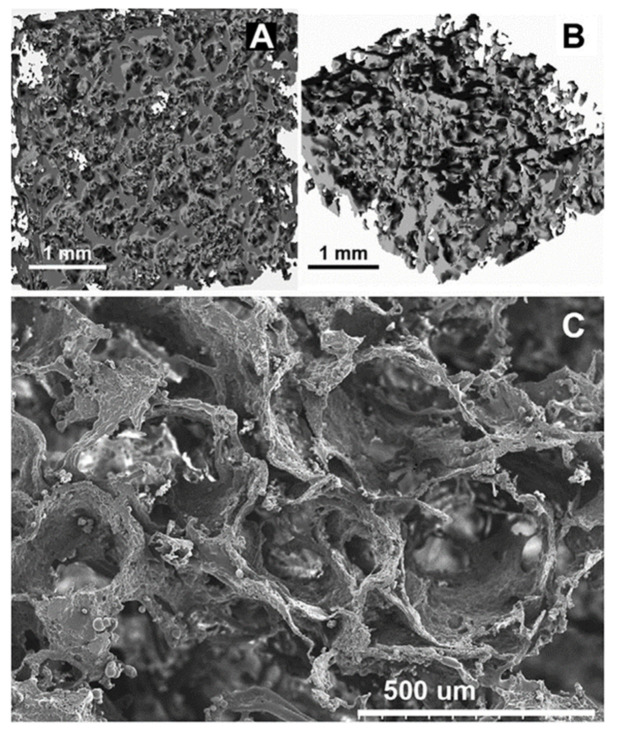
Polyurethane scaffolds. Micro-CT reconstructions: (**A**) top view, (**B**) 3-D view, (**C**) SEM image. Reprinted from [55] with permission from Elsevier.

Another approach to prepare PU elastomers was synthesis utilising IPDI and poly(ethylene adipate) diol. The reaction was carried out in the bulk and the obtained prepolymer chains were extended with hexamethylenediamine. For preparation of porous bone scaffolds the combined salt-leaching–phase-inverse technique was utilised. The scaffolds’ porosity achieved 83% with average pores size between 50 and 400 μm (Figure 6) [55]. It was also noted that of three different ranges in pore sizes (150–300, 300–500, 500–710 μm) the most robust cell growth was visible for the largest pores [107]. Polyurethane scaffolds obtained in this study exhibited a compressive strength equal to 0.16 ± 0.09 MPa [55].

Zuidema et al. [101] produced PU-based porous systems using 2-methyl pentane-diisocyanate (PDI) and sucrose, with the pore size distributions between 100 and 300 μm, as potential biomaterials. The authors found that this system will not be physically altered as the glass transition temperature was 67 °C, as well as pentane, which was used as a physical foaming agent, has been proved to be nontoxic and nonimmunogenic during in vivo tests. Additionally, sucrose is native to the body environment, undergoing transformation to fructose and glucose. It was observed that the dissociation of PDI–sucrose polyurethanes does not acidify the pH of the tissue environment. Biological test results suggested that the obtained PU does not induce an acute inflammatory or allergic reaction, biodegradation products are nontoxic, and material is biocompatible [108].

**Figure 7:**
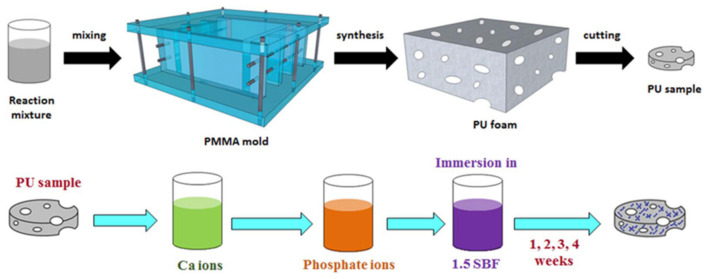
Schematic diagram of polyurethane foam/nano-hydroxyapatite composite fabrication. Reprinted from [57] with permission from Elsevier.

Polyurethane/nano-hydroxyapatite foam composites were also fabricated using bulk polymerisation and gas foaming process. In the synthesis, a prepolymer made of MDI and a mixture of polyether polyols was applied. A foam was obtained through mixing with distilled water as a blowing agent (Figure 7).

The biomineralisation on PU was initiated by immersion in CaCl_2_∙2H_2_O and wetting in Na_2_HPO_4_·12H_2_O to develop a layer of calcium phosphate. This was continued by the nucleation treatment of HAp in SBF. The samples subjected to biomineralisation displayed much better mechanical properties than the untreated one. For this reason, the polar character of PU is improved, and attachment of HAp enhances the mechanical properties of the composite. Polyurethane/nanoHAp foams exhibited properties close to mature bone due to the pore sizes and mechanical properties. An advantage is the ease of shaping during application. Longer treatment times (4 weeks) of the foam provided higher values of the elasticity modulus (25.40 ± 5.84 kPa) than for the untreated foam (13.75 ± 1.06 kPa) [57].

Giannitelli et al. [53] carried out studies on graded porous PU foams obtained in a one-step synthesis with water as a porogen. The foam scaffolds were prepared from PCL diol and polymethylenepolyphenyl isocyanate (PMDI) in two concentrations—13 and 19 wt.%. It was found that the loading of PMDI could impact on the mechanical properties and also on the foam architecture, especially porosity and pore size. An increase in the polyisocyanate content led to a lower Young’s modulus of 0.47 ± 0.29 and 19.54 ± 4.91 MPa, respectively, for 19 and 13 wt.% of PMDI.

Aguilar-Pérez et al. [109,110] investigated polyurethane-based foams modified with titanium. They prepared systems with castor oil (CO) and isophorone diisocyanate (IPDI) as well as with poly(ε-caprolactone) diol as soft segment and 4,4-methylene-bis cyclohexyl diisocyanate and l-glutamine as the rigid segment. It has been concluded that the addition of titanium particles had a beneficial effect on both human dental pulp stem cells and osteoblasts viability.

In the other paper by the same research group, PU glutamine-based segmented polyurethanes with 5–25 wt.% bioactive glass nanoparticles were prepared and it has been found that compositions with low content of bioactive glass nanoparticles are promising material for applications such as guided bone regeneration membranes [111].

In the next paper this team of authors investigated composites with HAp and segmented polyurethanes obtained with glutamine or ascorbic acid as chain extenders. SPUs were prepared with either l-glutamine or ascorbic acid as chain extenders. Semicrystalline PU with low glass temperature and elastomeric behaviour were obtained; biocompatibility investigations revealed that PUs containing ascorbic acid allowed the increase of alveolar osteoblast proliferation [112].

Luo et al. [113] investigated 3D porous PU/HAp scaffolds. The obtained PU/HAp composite with HAp content of about 30 wt.% exhibited porosity higher than 60% with pore size from 100 to 800 µm that is suitable for bone tissue regeneration. It has been observed that the obtained scaffolds degraded gradually in vitro showing clinically satisfying degradation time.

Lei et al. [114] obtained porous PU scaffolds and investigated the effects of water, isocyanate, and β-TCP on the selected physicochemical properties. They found that the adhesion strength of the obtained materials to bone was two times higher than acrylate bone cement. Moreover, the prepared scaffolds show, in in vitro tests, good biocompatibility.

### 5.2. Other Polyurethane Systems

PU/HAp materials are one of the most important groups of PU/ceramics composites. HAp [Ca_10_(PO_4_)_6_(OH)_2_] shows chemical similarity to human inorganic hard tissue. Both good interfacial adhesion between organic and inorganic phase and the homogeneous dispersion of ceramics at nano-level in the polymer matrix pose crucial factors for composites with bone-like properties [115]. The polymer matrix provides elasticity, compressive strength, integrity, and hardness. Regarding the other constituent, ceramics enhance the rest of the mechanical properties [116]. The bioactivity of composites is enhanced by the bioactive ceramics which interact with the surrounding bone. An ion exchange reaction between the composite scaffold and the body fluids leads to the formation of an apatite layer on the scaffold surface [117,118].

Perfect scaffolds are defined as a material made of resorbable polymers, biodegradable, and having a similar degradation rate to the growth rate of new tissues. Porosity is also important. It is needed for bone cells to build up in the scaffold and restore the porous structure, however, porosity significantly reduces the mechanical properties of such materials.

Biometric devices based on PMMA, including hard tissue implants, are used to restore tissue functionality. Unfortunately, their major disadvantages are the lack of integration into the host tissue, monomer toxicity, potential bone necrosis due to exothermic reaction, and inflammatory response. These undesirable properties have forced scientists to find another resorbable alternative to PMMA, which is, for example, injectable calcium cement with calcium phosphate. This material undergoes endothermic curing at a temperature of 37 °C. Another advantage is its resorbability and osteo-induction. However, this material has a significant disadvantage: its degradation rate does not correspond to the level of new bone formation. Materials that are actively involved in the healing process are still being sought, while at the same time they should exhibit mechanical properties comparable to that of the host bone. Due to their good biocompatibility, bioactivity, and ability to degrade in vivo by dissolution and osteoclastic resorption, calcium phosphates have been thoroughly tested as synthetic materials for bone implants. Polymer-ceramic composites have been considered as implants that can integrate with native bone and can carry considerable weight. Nonporous composites have been obtained through reactive compression moulding of mineralised allograft bone particles (MBP), with two-component biodegradable PU networks as the polymer binder, which is obtained from a polyester polyol and a lysine-derived isocyanate. PU can be used for injectable applications, i.e., bone cements. This is due to its two-component reactive liquid formulation. In addition, the polyurethane covalently binds to the filler of allogeneic bone by reacting isocyanate groups with collagen that occurs in the bone. Thanks to bone dimineralisation, the surface area that can react is increased. The mechanical properties increase due to the strong polymer bonding and filler phases. Due to the presence of the collagen layer, the adhesion of osteoblast-like cells is increased [67].

**Figure 8:**
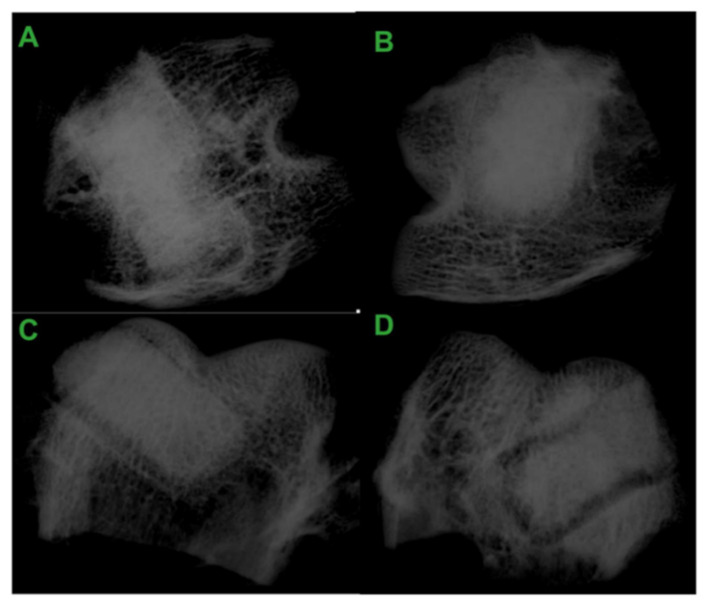
Radiographs of extracted rabbit distal femurs: (**A**) 6C3G1L300-MBP, (**B**) 6C3G1L300-SDBP, (**C**) 6C3G1L600-MBP, (**D**) 6C3G1L600-SDBP. Reprinted from [67] with permission from Elsevier.

An example of osteoconductive materials are MBP/PU composites, which exhibit high strength, thanks to which they are used for loading tasks. The rate of cell overgrowth and mechanical properties can be controlled by using different molar masses of the polyester segment. Cellular infiltration and bone formation in the interior of the implant occurs after 6 weeks. Osteoclast-mediated resorption of the allograft particles creates pores followed by cellular infiltration, the deposition of collagen matrix, and bone formation (Figure 8). The findings from the Dumas et al. [67] investigation suggest that MBP/PU composites show promise as biologically active, weight-bearing, bone substitutes.

Polymers may be used as natural biomaterials, synthetics, or a combination of both. A natural PU using polyester polyol obtained from Ricinus Communis, a tropical castor bean, has been synthesised as a potential material for bone regeneration. This PU resin exhibited lack of late inflammatory reaction and no signs of toxic effects. Histological examination of scaffolds inserted in rabbit skulls revealed bone tissue formation after 6 weeks. The incorporation of CaCO_3_ or Ca_3_(PO_4_)_2_ to the PU-based system improved the biocompatibility and osseointegration when implanted in rabbit tibia, as both additives are osteoconductive. In vitro studies with human osteoblasts revealed better cell adhesion, proliferation, and differentiation on Ca_3_(PO_4_)_2_. Barros et al. [54] evaluated the biocompatibility of three different types of PU systems with different chemical compositions. The results showed that all investigated implants were biocompatible. However, in vivo tests showed a wide range of responses like an inflammatory response, or no reaction. In vitro and in vivo biocompatibility of PU has been confirmed. In vitro studies showed that PU modified with calcium phosphate presented more bone-like formation than PU modified with calcium. In vivo tests revealed areas in which mineralised bone tissue was present at the PU–tissue interface. According to studies, the PU-systems chemical composition has no morphological influence on the PU–bone interface, but on the other hand, osseointegration was increased by the presence of CaCO_3_ at the 16th week, and by Ca_3_(PO_4_)_2_ after all evaluated periods. The osseointegration process was already complete on PU- Ca_3_(PO_4_)_2_ after 8 weeks.

Using the thermally induced phase separation technique, highly porous PUs can be obtained. Pore size distribution, porosity, and the morphology can be controlled by the PU content, quenching temperature, and the type of cosolvent. Optimal microstructures can be obtained at quenched temperatures of −25 and −15 °C, and by using 86:14 dioxane/water system. Such scaffolds coated with a layer of bone like apatite are promising bone substitutes due to their biocompatibility and high bioactivity [119].

Dias et al. [120] formulated porous scaffolds based on hydrolysable PU and montmorillonite (MMT). The insertion of MMT nanoparticles was expected to improve the selected mechanical properties. Scanning electron microscope (SEM) microphotographs showed interconnected pores with a size range of 184–327 μm. In vitro tests with osteoblasts demonstrated no cytotoxicity connected to the PU/MMT systems. In vivo results showed that cells fully colonised the entire implant through infiltration.

PU scaffolds were synthesised using IPDI, castor oil and BDO, while HAp was used as a bioactive component and uniformly dispersed in the PU. A 3D scaffold with interconnected pores was obtained by gas foaming. It was found that scaffolds with a HAp content from 20 to 60 wt.% could still hold with porosity up to 80% and had proper mechanical strength. The obtained PU/HAp scaffolds were characterised by a uniform HAp dispersion and a proper three-dimensional structure that makes them suitable for bone regeneration [121].

Carbon nanotubes (CNTs) were used to improve the selected properties and function of scaffolds. The incorporation of CNTs should also enhance the osteo-conductivity. Additionally, CNTs can be a structural reinforcement for the scaffold and introduce additional properties like electrical conductivity. The electric field is known to stimulate the healing and cell growth of various tissues through the physioelectrical signal transfer [122]. Zawadzak et al. [123] developed porous PU scaffolds electrophoretically coated with CNTs. It was found that the presence of carbon nanotubes on PU scaffolds enhanced the precipitation of a calcium phosphate phase. It was postulated that CNT’s negatively charged surfaces favour nucleation and mineralisation of HAp or calcium phosphate.

Vancomycin is an effective tricyclic glycopeptide antibiotic for curing infections caused by Gram-positive bacteria like *Staphylococcus aureus*. It is also less harming for osteoblasts and skeletal cells in comparison with other antibiotics, and it does not retard bone healing in vivo. Li et al. [42] incorporated vancomycin into PU scaffolds with tunable release kinetics over 6–8 weeks so that the processes of bone regeneration and formation were unconstrained by infection. PU scaffolds with different antibiotic release kinetics were evaluated to identify the most effective release strategy. PU scaffolds exhibited proper support and sustained in vitro release of antibiotic. It was revealed that PU scaffolds releasing vancomycin implanted in infected defects in rats significantly reduced the infection compared to the control without antibiotic.

For more than 20 years PU-based scaffolds or other devices have been widely used in many medical fields. PU-based materials have been used in intravenous catheters, cartilage replacements, vascular grafts, pacemaker lead insulation and artificial hearts. Moreover, PU materials can be applied with osteogenic growth factors and cells to improve the healing ability of surrounding tissues and can act as a scaffold for in vivo osteogenesis [124].

Boissard et al. [69] synthesised nanoHAp/PU composite scaffolds with polyester soft segments through the salt-leaching-phase inverse process. In vivo study revealed that the biocompatibility of the obtained scaffolds was not compromised by the nHAp incorporation. Asefnejad et al. [58] synthesised fluorohydroxyapatite (FHA)/PU scaffolds through solid–liquid phase separation. It was found that FHA nanoparticles improved the selected mechanical properties as well as the scaffold’s bioactivity. Investigations were carried out to determine the miscibility, chemical and mechanical properties, morphology, and water uptake of the composite samples. FTIR spectra proved the presence of hydrogen bonds between polyurethane and FHA particles. FHA increased the compressive strength and modulus of the PU scaffolds. It was concluded that samples with FHA content up to 20 wt.% exhibited suitable mechanical properties and porosity (80–88%). PU/Bioglass scaffolds with porosity over 70% and a pore size of 100–400 μm were described by Ryszkowska et al. [70]. Moreover, pores in scaffold wall (less than 10 μm) were found. Composite foams exhibited higher storage modulus in comparison to the unmodified PU scaffolds. The preliminary bioactivity assessment after incubation in SBF showed the high bioactivity of PU/Bioglass composites and the formation of apatite on the scaffold’s surfaces.

**Figure 9:**
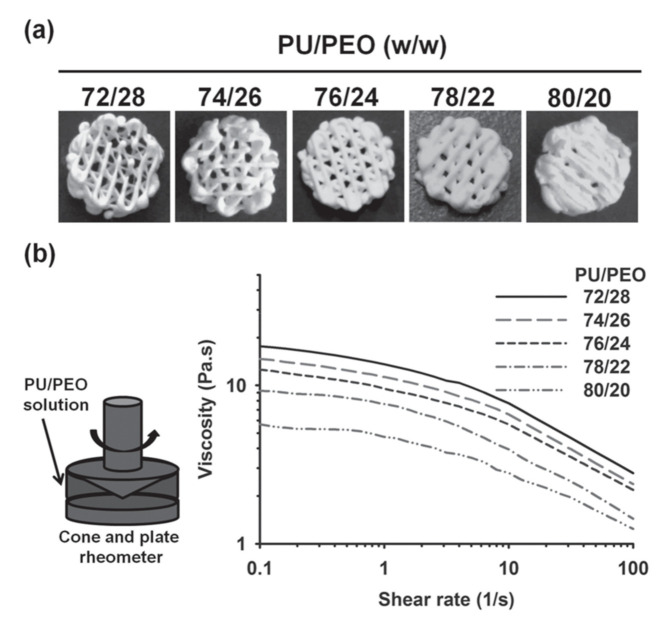
Fabrication of 3D scaffolds with various PU/PEO dispersions and rheological properties of the dispersions. (**a**) The gross appearance of the scaffolds fabricated with various PU/PEO ratios. (**b**) The viscosity of various PU/PEO dispersions in the shear rate range between 0.1 and 100 Hz. Reprinted from [127] with permission from Wiley.

PU-based blends and composites can be processed into filament, pellets or powders to enable 3D scaffolds printing using high temperature melting-extrusion and sintering as well as dissolved in organic/aqueous solvents to allow micro extrusion-based 3D printing at low temperature [125]. Hence, Cooke et al. [126] show that PU/PVA blends can be printed using themost 3D printing equipment to prepare 3D scaffold with promising properties for the craniofacial bone regeneration. Hung et al. [127] prepared biodegradable waterborne PUs with PCL and poly(ethylene-butylene adipate) (PEBA) diols as soft segments in the form of NPs in water-based process. Then the dispersion has been used to prepare 3D scaffolds by printing. Additionally, poly(ethylene oxide) (PEO) was used as viscosity enhancer (Figure 9).

The obtained scaffolds exhibited good potential for cartilage scaffolds development, as excellent seeding efficiency, proliferation, and matrix production of chondrocytes have been confirmed.

Shrestha et al. [128] obtained a porous osteopromotive fibrous scaffold from PU/Zein/CS-fMWCNTs system using electrospinning method. It has been found that the incorporation of carbon nanotubes in the PU composite membrane enhanced the biomineralisation and biodegradation. The membrane obtained exhibited good antibacterial efficacy and facilitated growth, proliferation, infiltration, and in situ differentiation of MC3T3-E1 cells in in vitro investigations.

Porous bone scaffold from PU, wintergreen (WG) and TiO_2_ was obtained by Jiang et al. who used a single-stage electrospinning [129]. The authors observed that the prepared scaffold showed improved wettability and thermal properties. It was concluded that enhanced calcium deposition and fibroblast proliferation make such systems promising material for bone tissue engineering.

### 5.3. Injectable Polyurethanes System

Designing and preparing synthetic PU as injectable systems, like gels or pastes, has been recently extensively studied. Injectable systems can be employed in minimally invasive treatments like an arthroscopy. Such systems could also be applied in complex bone fractures and in the repair of bone defects.

Another application field of the injectable systems is stabilisation and fixation of implants such as plates or screws, especially in patients with osteoporosis. The most desirable are systems where upon injection the liquid polymer, or prepolymer, will polymerise to the cured form without negative effects to the surrounding tissue like e.g., high temperature. It should also preserve the integrity and enhance cell attachment, proliferation, and growth. As yet very few injectable systems based on synthetic polymers with suitable properties for orthopaedic applications have been developed and applied. Two component injectable PUs useful as scaffolds for orthopaedic applications have been studied by Adhikari et al. [59]. Two-component injectable systems (prepolymer A and B) where star polyols based on lactic acid and glycolic acid, pentaerythritol (PE) and ethyl lysine diisocyanate (ELDI), have been prepared. Furthermore, the addition of water into the prepolymer leads to foam formation due to carbon dioxide release. PU systems were obtained by mixing components A and B. The obtained results exhibited high compressive strength and modulus. Moreover, it was revealed that incorporation of β-TCP improved the mechanical properties and retarded both in vitro and in vivo biodegradation process [59].

**Figure 10:**
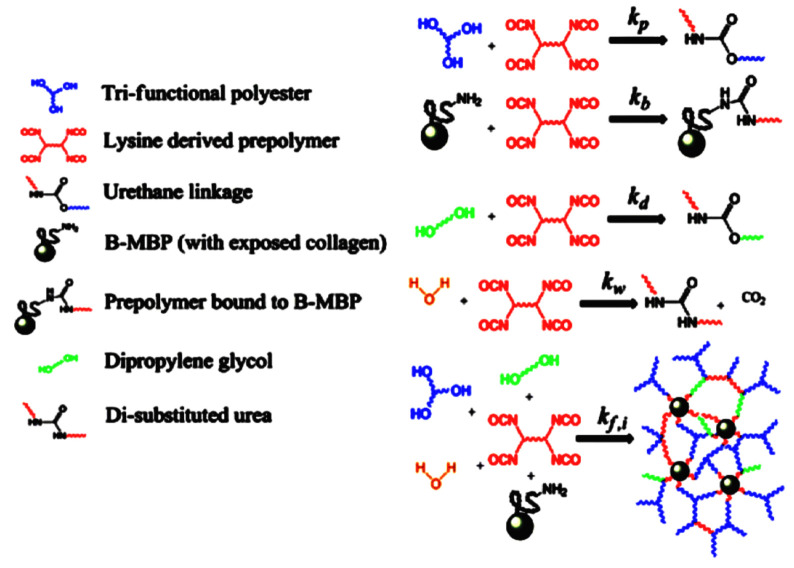
Chemical reactions present in the injectable PUR biocomposite. Reprinted from [130] with permission from Elsevier.

Pereira et al. [60] synthesised two injectable polyurethane acrylates (PUAs) using poly(propylene glycol) (PPG), or PCL diol and hydroxyethyl methacrylate. The PCL soft segments have ester groups that undergo hydrolysis, while the PPG soft segments in PU which are biostable, are more resistant to hydrolysis. One interesting example of injectable polyurethanes systems was synthesis with the addition of bovine mineralised bone particles (B-MBP), which were responsible for enhancing the mechanical properties of the composite and for stimulating bone tissue regeneration. The prepolymer based on lysine triisocyanate (LTI) and PEG (LTI-PEG) was mixed with B-MBP and catalysed by triethylenediamine (TEDA). The liquid PU obtained in this way is ready for injection to the bone defect. A process of cross-linking occurred under in vivo conditions. This strategy causes water diffusion from the surrounding tissues, enhancing the porosity and the material’s expansion. The curing in situ of PU-based injectable biocomposites occurs via five reactions occurring at the same time (Figure 10) [130].

## 6. Bioactivity and Biocompatibility of PU-Based Materials

**Figure 11:**
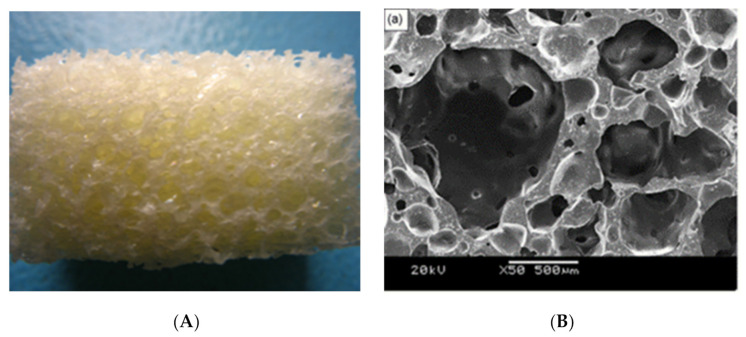
(**A**) Photo macrograph of the porous PU/HAp scaffold; (**B**) SEM photographs of the porous PU/HAp scaffold. Reprinted from [135] with permission from Elsevier.

Biocompatible should not have a negative influence on the host tissue and immune system. The toxicity of biomaterials leads to inflammatory reaction in surrounding tissues, haemolysis induction, cell damage, or the generation of free radicals that can affect other tissues or organs. Ohara et al. [131] inserted PU cylindrical implants obtained using castor oil into rabbit bones. Biological tests did not show cell degeneration in pathological changes in the animal’s organs after 40 days after implantation. New formed bone tissue derived from fibrous tissue around the PU implant and in the implant pores at different levels of calcification was detected and assessed. As in the material of the present work, no antigen or inflammatory reaction was detected [131,132,133,134]. Moreover, after the PU curing, no damage of the surrounding tissues was observed [134]. A porous PU/HAp biomaterial was obtained using castor oil. PU with a castor oil crosslinker exhibited a good degree of crosslinking as well as good foaming ability at the desired temperature. The authors obtained PU scaffolds with high porosity and interconnected pores which is desirable for cell adhesion, proliferation, and growth (Figure 11).

The compressive strength and degradation property are suitable in tissue engineering for cartilage tissue implants. The obtained scaffold exhibited proper in vitro cytocompatibility and in vivo biocompatibility [135].

PU and PU/PLDLA based macroporous composites were obtained using the slurry-dipping technique to coat scaffolds with bioglass. Bioactive glass coating enhances the formation of a carbonate hydroxyapatite layer on the scaffold’s surface which is a sign of high bioactivity [136].

In order to increase the PU bioactivity, various surface modifications were applied. One proposed modification was to incorporate peptides such as fibronectin or collagen. For the application of PU for bone tissue engineering, peptide grafting, plasma gas treatment, or collagen immobilisation (most often applied) have been used [137].

## 7. The Influence of Polyurethane Materials on Cell Proliferation and Differentiation

Segmented PU elastomers consist of structurally linear alternating block copolymers of polar hard and nonpolar soft segments. Glass transition temperature of the soft segment is typically below 0 °C. Hydrogen bonds between urethane and/or urea groups leads to the formation of semicrystalline hard domains which act as physical cross-linkers when the material is under mechanical stress and allows it to keep its shape or to behave like shape-memory systems. They can be disrupted above the hard segments melting point or by dissolving in an aprotic solvent. The chemical structure of the hard and soft segments and their ratio, as well as type of chain extenders/crosslinkers, determine the degree of microphase separation and, in consequence, the mechanical properties. Generally, using diamine chain extenders leads to the formation of urea bonds and the formation of hard segments with higher melting temperatures in comparison with polyurethanes prepared from diol chain extenders. With an increase of the chain extender/crosslinker to polyol molar ratio, the content of hard segments also increases, and the obtained PU is harder and stiffer but is characterised by a lower elongation at break. On the other hand, the degradation rate is affected by the soft segment composition. Hence, PU with soft segments based on PCL-*b*-PEO-*b*-PCL block copolymers undergo faster hydrolytic degradation than PU with PCL-based soft segments. Such an effect was explained by the higher hydrophilicity of the PCL-*b*-PEO-*b*-PCL block copolymer with hydrophilic PEO units. PU scaffolds have been obtained from biodegradable segmented elastomers using thermally induced phase separation, salt leaching/freeze drying, wet spinning of fibres, and electrospinning of fibres. For filling of large bone defects, PU scaffolds with a very slow biodegradation rate have to be used to prevent tissue collapse and to sustain newly forming tissue. In this regard, the range of properties in the case of PUs is notably larger than with commonly used medical polymers. Mesenchymal stem cells (MSC) pose an effective alternative to primary autologous bone cells and they can be harvested from adult tissues. Adequate chemicophysical, morphological, and mechanical properties of synthetic or natural polymers might boost the activity of MSCs in guiding tissue neoformation. PU foams show not only good morphological and mechanical properties, but also stimulate cell adhesion, proliferation, and differentiation of MSCs which renders them good candidates as bone graft substitutes. Incorporation of bioactive fillers or coatings might improve the performance of MSCs on PU-foamed scaffolds [1].

Mrówka et al. investigated the effect of various catalysts like DBTDL and *N*-dimethylethanolamine (*N*-met) on cell viability in poly(siloxane-urethaneoureas) synthesised using IPDI. The test was performed using the XTT protocol on days 7 and 21 of culturing. The number of cells on the PU surface without catalyst was much higher than on the control culture on polystyrene. However, the highest lifetime was found on PU with the addition of *N*-met, and the worst result was obtained by the DBTDL catalyst [134,137]. Podporska-Carroll et al. [55] investigated the interaction between cells MG63 and poly(ester-urethane) urea three dimensional scaffolds obtained using IPDI, poly(ethylene adipate) diol (PEA) as a polyol, and hexamethylenediamine (HDA) as a chain extender. Thanks to scaffolding, MG63 cells were provided with better growth and proliferation. The samples were subjected to stimulation with osteogenic phosphatase, which after 2 weeks reached the maximum and remained at this level. In SEM analysis, calcium phosphates deposits were observed on scaffolds covered with cells [137]. Bil et al. [136] conducted studies on the impact of rigid segments on the properties of PU surfaces, in particular hydrophilicity. Human bone-derived cell (HBDC) was used for the study, which was cultured on PU containing from 22% to 70% by weight of rigid segments. It has been proved that the higher the content of rigid segments results in an increased hydrophilicity of the PU surface. The highest cell viability has been revealed on the most hydrophilic PUs. In studies by Kommareddy et al. [138] various PUs with PTMG soft segments were synthesised with HMDI, BDO, and used as a model for an impact study of scaffold stiffness in cell proliferation (MC3T3-E1) and bone formation. The smallest PU had the smallest number of cells compared to stiffer samples. It was found that high stiffness of the material affects cell proliferation. Mrówka et al. studied the stiffness of scaffolds using PU films on cancer cells and came to similar conclusions [17,138,139]. In another work, Kuo et al. [139] discussed the behaviour of human mesenchymal stem cells (hMSC), which were applied to PU fibres with different elasticity. The fibres were obtained by electrospinning (PCL-IPDITEA-EDA) on a PCL electrolytic scaffold. Studies have shown that the material hardness has a significant effect on cell proliferation. As the elastic modulus increases, osteogenesis increases while still maintaining the same size of fibres. However, fibres with smaller diameters enhance more cell differentiation than fibres with larger diameters [137,139]. Irving et al. [140] in their work compared the method of processing polyurethane material that serves as a cellular substrate. The results show that both random grinding and laser machining can be used to control the behaviour of cells. The conclusion of the work is the fact that in order to increase cell adhesion and their proliferation, it is possible to apply polishing which results in functional surfaces with directional or random features at the nanoscale.

## 8. Biodegradation of PU Scaffolds

**Figure 12:**
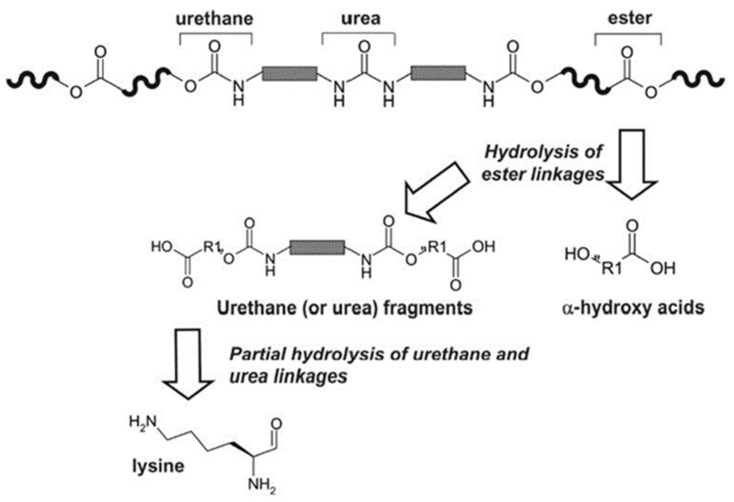
Postulate mechanism of PU biodegradation. Reprinted from [23] with permission from Elsevier.

The urethane bonds undergo hydrolysis with difficulty. That is why the biodegradation rate is determined by the soft segment’s chemical structure with ester groups derived from polyesters. Chain extenders/crosslinkers with hydrolysable ester linkages allows PU hard segments to be biodegradable. According to preclinical models of tissue repair, PEUs undergo degradation to noncytotoxic products and enhance the ingrowth of new bone tissue. PEU scaffolds based on LDI degrade faster in vivo in comparison with in vitro conditions. PEU scaffolds prepared from LTI or trimer of 1,6-hexamethylenediisocyanate (HDIt) undergo hydrolytic, esterolytic, and oxidative degradation. Hydrolysis of ester bonds in the PU structure to low molecular α-hydroxy acids takes place for the most part in PBS (Figure 12). The biodegradation rate slightly increases in esterolytic media. It has been found that LTI-based scaffolds degrade six times faster in an oxidative medium than HDIt-based scaffolds. In general, in vitro degradation of the scaffolds yields water-soluble degradation products that, in vivo, might be easily eliminated from the body [141].

Waterborne PU or PUU formulations are attracting an increasing interest as they are characterised by low viscosity at high molar mass, nontoxicity, and good applicability, not to mention their easier biodegradation. Natural oil polyols derived from vegetable oils (e.g., grapeseed oil, castor oil) are also used in the production of waterborne PUs, thus rendering them nature friendly materials [43].

Degradation tests of the block copolymer poly(ethylene glycol)-poly(serinol hexamethylene urethane) (ESHU) have been performed in pure PBS and cholesterol esterase (CE) solution. It was observed that the presence of CE accelerated the degradation. PUs undergo biodegradation by enzymes as well as by the oxidative activity of the immune system, and the biodegradation rate of ESHU is more intensive in vivo [142].

Due to their excellent physical properties and biocompatibility, PU elastomers can be classified as one of the most commonly used biomaterials. Their main use as medical devices are blood filters, catheters, heart valves, stimulating electrodes, or devices supporting the work of the heart. Polyurethanes are most often made of oligomeric diol, diisocyanate, and a chain extension, which suggests potential toxic degradation in the biological environment. Unfortunately, in some cases it has been documented that degradation of PU leads to the release of toxic products. Therefore, the use of PU in long-term implantation has been limited.

PUs can be obtained by the synthesis of aromatic diisocyanates. One of them is toluene diisocyanate (TDI). Studies have shown that such polyurethanes can hydrolyse in both acidic and alkaline environments, or at high temperatures, releasing toluene diamine (TDA) with a carcinogenic effect. Chan et al. conducted in vitro studies that confirmed that TDI-based PU hydrolyses with the release of TDA. The authors examined urine samples taken from women with polyurethane breast implants. Unfortunately, before analysis, the samples were subjected to hydrolysis at 105 °C in an acid medium. Detection of TDA and its derivatives is necessary to assess PU toxicity. Unfortunately, little research has been conducted in this direction so far.

In the work by Labow and Santerre [143,144], the authors noted that cholesterolesterase (CE) is a helpful enzyme in catalysing the hydrolytic degradation of TDI-based PU. CE occurred mainly in the monocytes due to the fact that they differ in macrophages at implantation sites [17]. The same authors have signalled in an earlier work that other compounds are released during the incubation of PU with CE [18]. Santerre et al. undertook the analysis of the products of this incubation [144]. Unfortunately, their actions were hampered by protein impurities that caused incomplete separation of degradation samples. Deproteinisation of samples is necessary before analysing biodegradable products containing protein. This process is very important, especially when reverse phase high performance liquid chromatography (HPLC) is used [21,22]. In addition, proteins have the predisposition to aggregate in gradient analysis mode, which later results in their falling out during the gradient run [23]. In previous works on the separation of polyurethane biodegradation, it was found that classical methods of deproteinisation are not sufficient to remove proteins contained in samples. An additional risk is the fact that the addition of a base or acid can lead to further hydrolytic degradation of the degradation products. However, there is an alternative method that gives satisfactory results. It is a method using Ultrafree (UF) filter units with different molecular weight cutoffs. By using this method, it is possible to remove high molecular weight proteins and related residues [27]. However, solutions are still being sought that will give even better results. Scientists believe that combining the UF method with classic deproteinisation techniques will allow better separation of biodegradable products. The article describes an analytical method for the isolation of biodegradation products that came, in particular, from PUs with inflammatory enzymes. The method is based on reverse-phase HPLC separation combined with tandem mass spectroscopy (MS-MS) for identification. Labelled PU t4C was synthesised and used to confirm the product discovery.

As mentioned earlier, polyurethane elastomers are one of the most important groups of polymers for biomedical applications. This use is due to their physical properties and biocompatibility. Unfortunately, due to their potentially toxic degradation in the biological environment, the use of PU as long-term implants raises controversy. In addition, based on studies confirming the release of potentially toxic compounds during PU degradation, the use of polyurethanes for long-term implantation has been limited [145].

There were no clear signs of oedema in the lungs exposed to MDI-based PU foam. The concentration of MDI is four times higher at 350 °C than at 450 °C because both particulate and gaseous isocyanates were found in the former case, whereas only gaseous isocyanates were determined in the latter. Consequently, the same broncho constriction was obtained at both 350 and 450 °C. The thermal degradation products from TDI-based PU foam did not cause any deterioration in lung function. The enzyme and fungi degrade each PU. It has also been found that increases in the chain length of the polyesters affects their biodegradability [146]. Phua et al. [147] observed that a medical polyester PU might undergo degradation under the influence of papain and urease. Urease activity was limited to the scaffold surface due to its molecular weight of 473 kDa. The authors proposed that papain hydrolyses the urethane and urea linkages by exuding free amine and hydroxyl groups. On the other hand, for polyether PU, both papain activity and aqueous hydrolysis results in the release of biodegradation products. Water leads to hydrolysis of the ether linkages while degradation of the urethane groups is connected with the presence of proteolytic enzyme. Labow et al. [143] treated PU, with both polyester and polyether-based soft segments, with human neutrophil elastase and porcine pancreatic elastase. It was found that porcine pancreatic elastase caused a 10 times higher PU degradation rate than human neutrophil elastase. Moreover, the degradation of PU with polyester soft segments by porcine pancreatic elastase is 10 times faster in comparison with polyether PU. Akutsu et al. [148] postulated that PU degrades under polyurethanase enzyme in a two-step reaction. At the beginning, the hydrophobic adsorption onto the PU surface followed by the hydrolysis of the PU ester bonds take place. The PU esterase consists of a catalytic domain and a hydrophobic-PU-surface binding domain (SBD), which is essential for biodegradation. The same structure has been reported in polyhydroxyalkanoate (PHA) depolymerase. So far, only two types of polyurethanase enzymes have been isolated and described: membrane bound PU-esterase and soluble, extracellular PU-esterases. The two types of polyurethanase seem to have different roles in biodegradation which would be beneficial for the PU-degrading bacteria. Hydrolysis is possible thanks to attachment of the bacteria cells to the polyurethane substrate via the PUase, followed by secretion of soluble metabolites. In turn, extracellular PU-esterase hydrolyses the polymer thus allowing for the metabolism of soluble products and facilitating access for enzymes to the initially degraded polymer [149].

**Figure 13:**
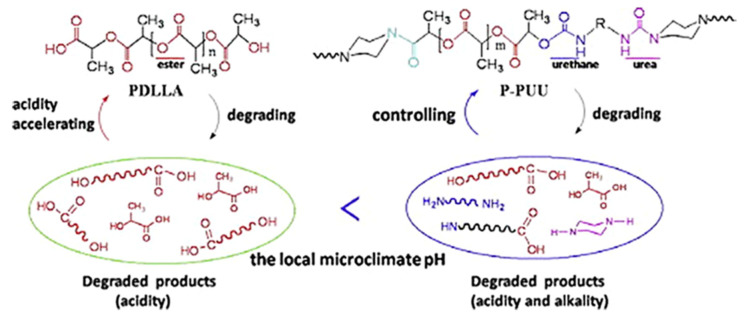
The degradation of PLA and P-PUUs. Reprinted from [137] with permission from Elsevier.

In another work, PU was subjected to a controlled degradation. The PU with soft segments consisted of 60% glycolide, 30% _D,L_-lactide, and 10% PCL diol, LDI, pentaerythritol as a crosslinker, and organic bismuth compounds as a catalyst (Figure 13). Thanks to the use of these substrates, the PU degradation products influenced the pH of the environment. Another team of researchers (Ruan et al.) used HDI, PLA and PP pipazine to produce PPUU. The use of PP improves the hydrophilicity of PPUU and can eliminate acidic PLA products, thanks to its alkalinity [137].

Another oligodiol used for PU production is PEG. PEG is nontoxic, and soluble in water and organic solvents. The final studies indicated that it increases the degree of PU degradation [137]. PEG increases the total hydrophilicity and the ability to absorb water into the material, which accelerates the hydrolysis of the ester bond and the faster release of degradation products [137]. The choice of isocyanates is also very important. Aliphatic or cyclic diisocyanates such as HDI, BDI, HMDI, or IPDI, are degraded to nontoxic products [137,150].

## 9. Conclusions and Future Trends

The crucial point in preparation of novel PU-based systems for bone tissue regeneration is elaboration of material composition that leads to biocompatible polymers still maintaining proper physicochemical properties. Moreover, scaffold preparation method shall enable its application during surgery.

The use of injection scaffolds minimises injuries and pain accompanying operations. Potentially, the use of alignment of the cavity geometric shape and in situ polymerisation can lead to the minimisation of invasive procedures. This is related to the low mechanical strength of these materials and the products of acid degradation. Recently, a two-component injectable polyurethane-based material with β-tri-calcium phosphate granules has been developed. Thanks to the use of TCP, this material exhibited more favourable mechanical properties compared to existing injectable materials, further preserving the proliferation and viability of bone cells in vitro. The missing studies regarding this material are studies on its ability to promote bone cell differentiation and an in vivo test. Despite the lack of research, this material is a promising alternative to bone grafts. The material applied for implantation should improve cell attachment and viability, as well as proliferation to induce osteogenesis. For this purpose, hybrids of polymer and HA or TCP have been developed. Moreover, to enhance this effect, human cells are incorporated. However, cell attachment and viability depend on the catalyst complex. The subsequent factor is the rate of hydrolysis of the implanted material. In this case, large bone defects require a slow rate of degradation due to supporting new formed tissue. Thus, there is a need to bridge the ability for cell proliferation and the good biodegradation abilities.

## Figures and Tables

**Table 1:** Substrates used in biomedical polyurethane synthesis.

Isocyanates			Polyols			Chain Extenders/Crosslinkers		
**Name**	Structure	Ref.	Name	Structure	Ref.	Name	Structure	Ref.
HDI	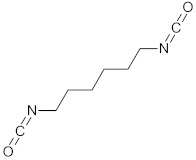	[46,49,50]	PEG, PEO	−[CH_2_−CH_2_−O]_n_−	[49,50,51,52,53]	1,6-hexamethylenediamine (HMDA)	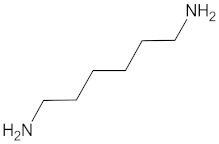	[44]
LDI	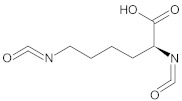	[14]	PCL diol	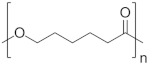	[12,23,49,51,54]	Tyramine (Tyr)	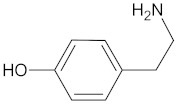	[23]
MDI	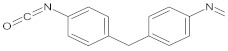	[1,4,21,44]	PEA diol	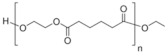	[55]	2,2,3,3-Tetrafluoro-1,4-butanediol (TFBD)	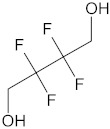	[44]
HMDI	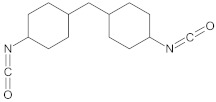	[12,54]	PC diol	-	[51]	1,4-butanediol (BD)	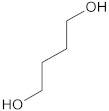	[44]
BDI	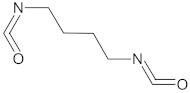	[6,49,51]	PLA diol	HO-PLA-OH	[56]	*N*-methyl diethanoloamine (N-MDA)	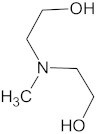	[44]

**Table 2:** Polyurethanes investigated for biomedical applications.

Polyol	Isocyanate	Chain Extender/Crosslinker	Additives (Content)	Young Modulus (GPa)	Compressive Strength (MPa)	Tensile Strength (MPa)	Comments	Ref.
PEA	IPDI	Hexamethylene diamine	n/a	n/a	0.16	n/a	Poly(ester urethane) urea elastomer was synthesised in bulk; The scaffolds were prepared by a combined salt-leaching-phase inverse technique	[55]
Polyether	MDI	n/a	n/a	0.0000254	n/a	n/a	Process consisted in a one-step bulk polymerisation, performed by gas foaming reaction	[57]
PCL diol	Polymethylene polyphenyl isocyanate (PMDI)	n/a	n/a	0.019	n/a	n/a	One step synthesis. Foaming with water in polyethylene mould	[53]
PCL diol	HDI	BDO	Nano fluor-hydroxyapatite (nFHA) (5–20 wt.%)	n/a	0.36–0.61	n/a	The polyurethane was obtained in a two-step process; nanocomposite foams were prepared by solid–liquid phase separation, subsequent freeze drying	[58]
Pentaerythritol/glycolic acid (PE/GA)	ELDI	n/a	n/a	n/a	136	n/a	Two-part injectable prepolymer systems (prepolymer A and B);	[59]
Pentaerythritol/glycolic acid (PE/GA)	ELDI	β-TCP (10 wt.%)	n/a	n/a	139	n/a		
Pentaerythritol/PDLLA	ELDI	n/a	n/a	n/a	145	n/a		
Pentaerythrito/glycolic acid/PDLLA	ELDI	β-TCP (10 wt.%)	n/a	n/a	187	n/a		
Pentaerythrito/glycolic acid/PDLLA	ELDI	n/a	n/a	n/a	179	n/a		
Pentaerythrito/glycolic acid/PDLLA	ELDI	β-TCP (10 wt.%)	n/a	n/a	104	n/a		
PPG	IPDI	Camphorquinone (CQ), 2-(dimethylamino)ethyl methacrylate (DMAEM)	n/a	0.00084	n/a	1.04		[60]
PCL diol	IPDI	Camphorquinone (CQ), 2-(dimethylamino)ethyl methacrylate (DMAEM)	n/a	0.01	n/a	6.45	Polyurethane acrylate synthesis; Preparation of PUA films used photoinitiator and co-catalyst for curing	
PEG	HDI, MDI	BDO, chitosan, starch	0–15% HAp	n/a	n/a	n/a	PUs were synthesised in bulk by a two-step polymerisation method with starch or chitosan as crosslinker	[61,62,63]
PCL diol	HMDI	Ethylene glycol (EG)	n/a	0.039	n/a	n/a	PUs were synthesised in bulk by a two-step polymerisation method	[52]
PEG 1000 PCL diol	BDI	Putrescine	n/a	n/a	n/a	8.0	The synthesis process of poly(ether ester urethane)urea (PEERs) was conducted under a dry nitrogen atmosphere in a two-step solution polymerisation	[51]
PEG 2000	HDI	Gold nanotube/nanowire			0.26 (at 37 °C)		For fabrication of porous scaffolds, 355–600 μm sieved table salt was added to the remaining solution, and the mixture of polymer and salt was casted in the Teflon mould having a 10 mm diameter	[49]
PEG 6000	HDI		Calcium stearate	0.001			Foam formation started 10–15 s after introduction the catalyst and stopped after 90–100 s due to crosslinking of polymer	[64]
PCL diol 2000 PEG 1000	BDI		Putrescine			1.22		[65]
PCL diol	1,4-butanediisocyanate	BDA, BDO	n/a	n/a	n/a	145	In the case of chain extension with a longer urethane diol block, a processible polymer was obtained with mechanical properties comparable to the polyurethane urea	[66]
PCL diol	BDI	Tyramine (TyA)	n/a	0.278	n/a	n/a	The PCL macrodiol were obtained from a BDO initiator and ε-caprolactone monomer	[23]
PCL diol	LTI		n/a	n/a	3–6 GPa	107–172 MPa	Nonporous composites have been fabricated by reactive compression moulding of mineralised allograft bone particles	[67]
PCL diol	HDI	Isosorbide diol (1,4:3,6--dianhydro--D--sorbitol) (Iso)	n/a	n/a	0.8 MPa	n/a	A two-step combined bulk-solution polymerisation was applied to synthesise the polyurethane used in the study	[68]
PCL diol	HDI	1,4,3,6-Dianhydro-d-sorbitol (ISO)	Nanohydroxyapatite	0.00126	n/a	n/a	The PU was synthesised in a one-step solution polycondensation	[69]
PCL diol	HMDI	Ethylene glycol (EG)	Bioglass^®^	n/a	n/a	n/a	Porous scaffolds were fabricated by polymer coagulation combined with the salt-particle leaching method	[70]

## Data Availability

Data sharing not applicable.

## Abbreviations

BDA	1,4-butanediamine
BDI	1,4-diisocyanatobutane
BMSC	bone marrow stromal cells
CE	cholesterol esterase
DMA	dynamic mechanical analysis
DSC	differential scanning calorimetry
ELDI	2,6-diisocyanato hexanoate
ESHU	ABA block copolymer, poly(ethylene glycol)-poly(serinol hexamethylene urethane)
HAp	hydroxyapatite
HDI	1,6-hexamethylenediisocyanate
HDIt	trimer of 1,6-hexamethylenediisocyanate
LDI	lysine diisocyanate
LTI	lysine triisocyanate
MDI	4,4′-diisocyanate diphenylomethane
MSC	mesenchymal stem cells
PBS	phosphate buffered saline
PC diol	polycarbonate diol
PCL	poly(ε-caprolactone)
PEA	poly(ethylene adipate) diol
PEG	poly(ethylene glycol)
PEU	poly(ester urethane)
PEUUR	poly(ester urethane)urea
PEEUUR	poly(ether ester urethane)urea
PGC-diol	poly[glycolide-co-(ε-caprolactone)]-diol
PHA	polyhydroxyalkanoate
PHB	poly[(R)-3-hydroxybutyrate]
PLLA	poly(l-lactide)
PLGA	poly(lactide-co-glycolide)
PMMA	poly(methyl methacrylate)
PPF	poly(propylene fumarate)
PPG	poly(propylene glycol)
PSU	polysulphone
PTMC	poly(trimethylene carbonate)
PU	polyurethane
PUU	poly(urethane-urea)
PVLCL	poly(δ-valerolactone-co-ε-caprolactone)
RP	rapid prototyping
SEM	scanning electron microscope
TCP	tricalcium phosphate
TEDA	triethylenediamine
TIPS	thermally induced phase separation technique
TMHDI	2,2,4-trimethyl hexamethylene diisocyanate
TyA	tyramine
WAXS	wide-angle X-ray scattering
